# The Ubiquitin System: An Emerging Therapeutic Target for Lung Cancer

**DOI:** 10.3390/ijms22179629

**Published:** 2021-09-06

**Authors:** Jun-O Jin, Nidhi Puranik, Quyen Thu Bui, Dhananjay Yadav, Peter Chang-Whan Lee

**Affiliations:** 1 Shanghai Public Health Clinical Center & Institutes of Biomedical Sciences, Shanghai Medical College, Fudan University, Shanghai 201508, China; 2 Department of Medical Biotechnology, Yeungnam University, Gyeongsan 38541, Korea; 3 Biological Sciences Department, Bharathiar University, Coimbatore 641046, Tamil Nadu, India; nidhipuranik30@gmail.com; 4 Department of Biomedical Sciences, University of Ulsan College of Medicine, Asan Medical Center, Seoul 05505, Korea; quyenbt86@gmail.com

**Keywords:** lung cancer, ubiquitin, ubiquitination, E3 ligase, cell signaling, deubiquitination

## Abstract

The ubiquitin system, present in all eukaryotes, contributes to regulating multiple types of cellular protein processes such as cell signaling, cell cycle, and receptor trafficking, and it affects the immune response. In most types of cancer, unusual events in ubiquitin-mediated signaling pathway modulation can lead to a variety of clinical outcomes, including tumor formation and metastasis. Similarly, ubiquitination acts as a core component, which contributes to the alteration of cell signaling activity, dictating biosignal turnover and protein fates. As lung cancer acquires the most commonly mutated proteins, changes in the ubiquitination of the proteins contribute to the development of lung cancer. Various inhibitors targeting the ubiquitin system have been developed for clinical applications in lung cancer treatment. In this review, we summarize the current research advances in therapeutics for lung cancer by targeting the ubiquitin system.

## 1. Introduction

### 1.1. Lung Cancer

According to the World Health Organization (WHO), cancer is the primary cause of premature deaths, accounting for about 30% of deaths from noncommunicable diseases [1]. Lung cancer comprises about 11.6%, breast cancer 11.6% and colon cancers at approximately 10.2%. Lung cancer is the major cause of death from cancer (18.4% of all deaths), followed by colon cancer (9.2%) and gastric cancers (8.2%) [2,3]. 

**Figure 1:**
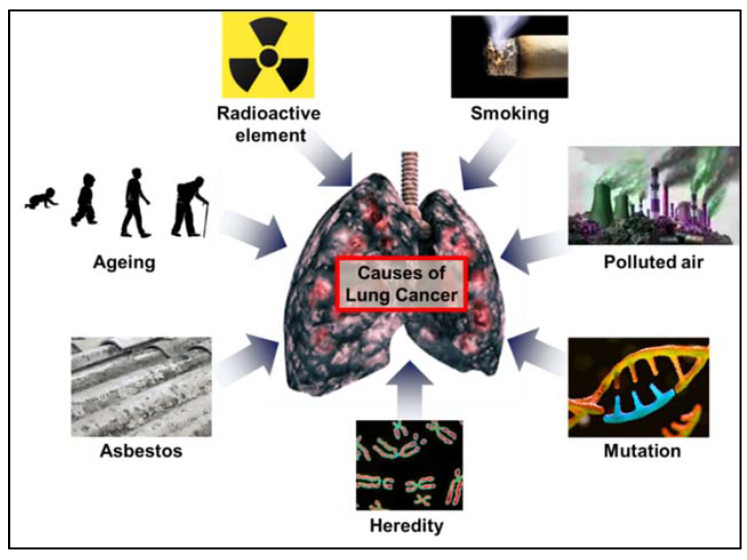
Common risk factors for lung cancer.

Lung cancer is the world’s leading cause of cancer deaths because it is initially asymptomatic and is usually only diagnosed at advanced stages [4]. Lung cancer is caused by various factors, as shown in Figure 1. Smoking, polluted air, radioactive elements and asbestos are common causative factors of lung cancer, while mutations, heredity and aging are also responsible for the development of lung cancer. Smoking is the number one cause of lung cancer; however only approximately 15% of cigarette smokers suffer from lung cancer, suggesting a genetic vulnerability. In support of this, lung cancer is also associated with family history [5]. Chronic obstructive pulmonary disease (COPD) commonly leads to the development of lung cancer and the treatment of COPD-associated lung cancer is very challenging [6].

Lung cancer comes in many subtypes, and the most defined subtypes are small cell lung carcinoma (SCLC) and nonsmall cell lung carcinoma (NSCLC) [7]. NSCLC comprises about 85% of total lung cancer cases, while SCLC makes up around 15% of all cases [8]. A basic comparison of SCLC and NSCLC is given in Table 1.

### 1.2. Small Cell Lung Cancer (SCLC)

SCLCs usually originate in a peribronchial location with infiltration of the bronchial submucosa and peribronchial tissue [9]. Bronchial obstruction is usually caused by circumferential compression, but endobronchial lesions can occur in rare cases. It is uncommon to find SCLC in a surgical specimen because the diagnosis is generally made through transbronchial biopsy or cytology. Widespread lymph node metastases are frequent. The tumor is white-tan, soft, and friable, with extensive necrosis. Extrinsic compression may obstruct the bronchial lumen in the advanced disease. In up to 5% of cases, SCLC manifests as a single solid lesion. In the 1981 WHO classification, three subtypes of SCLC were proposed: (1) oat cell carcinoma, (2) intermediate cell type, and (3) combined oat cell carcinoma [10]. 

Most SCLC cases demonstrate a loss of cyclin dependent kinase inhibitor 2A (CDKN2A) with mutations in *TP53* and *RB1*, a loss of PTEN and activation of PI3K [11,12]. C-myc is activated through the activation of cell cycle driving proteins, an increase in antiapoptotic proteins and metabolic modulation. It is a transcription factor and a major effector molecule in cellular signaling, and regulates lung cancer cell characteristics such as growth, resistance, death, and dissemination [13]. The heterogeneity of lung cancers makes it difficult to understand the mechanisms of specialized agents that act differently in a diverse patient population [14]. 

### 1.3. Nonsmall Cell Lung Cancer (NSCLC)

NSCLC is the most common type of lung cancer. NSCLC has been further classified by the WHO into three main subtypes: adenocarcinoma, lung squamous cell carcinoma (LUSC), and large cell undifferentiated carcinoma [15]. Among the three subtypes of NSCLC, LUSC is the most common type; however, the molecular mechanism underlying this type of cancer is still unclear [16].

Many molecular changes, including oncogenes and tumor suppressor genes, have been discovered in NSCLC in recent years, and many of them provide novel prognostic biomarkers or targets for cancer therapy [17]. The chief signaling pathways that may provide roadmaps for lung cancer therapy are growth-stimulating pathways (EGFR/Ras/PI 3-kinase), growth inhibitory pathways (Rb/p53/P14ARF/STK11), apoptotic pathways (Bax/Bcl-2/FasL/Fas), DNA repair and immortalization genes [18].

## 2. Ubiquitination and Deubiquitination

The UPS (ubiquitin–proteasome system) is a specialized proteolysis system that regulates protein degradation and is critical for cellular protein homeostasis. The UPS is made up of several key components, including ubiquitin, ubiquitin-activating enzymes (E1s), ubiquitin-conjugating enzymes (E2s), ubiquitin ligases (E3s), deubiquitinating enzymes (DUBs), and the 26S proteasome [19]. The E1 enzyme in the UPS is in charge of activating ubiquitin molecules, and various drugs have been discovered to be E1 inhibitors. E1 enzymes have two ubiquitin activating enzymes referred as UAE (well known as UAE1) and UBA6. These are responsible for initiating the ubiquitin conjugation in mammals. UAE and UBA6 are involved in activating cellular ubiquitin, an estimated >99% and 1%, respectively [20]. The E2 enzyme attaches to E1, and the activated ubiquitin is then transported from the E1 enzyme to a cysteine in the E2 enzyme. Subsequently, the E2 enzymes play a role in ubiquitin conjugation to substrates. Several E2 inhibitors have been discovered to obstruct the process in recent years [21,22]. The 26S proteasome is an ATP-dependent multisubunit complex that hydrolyzes proteins into tiny peptides. It is made up of one 20S core particle (20S CP) and one or two 19S regulatory particles (19S RP) [23]. 

Ubiquitination is the most common mechanism among the post-translational modifications (PTM) of proteins that regulate various cellular processes in distinctive manners [24]. In these processes, ubiquitin (Ub), a highly conserved 76-amino-acid protein that is expressed in all eukaryotic cells, performs various cellular functions by conjugation to other cellular proteins to regulate them [25,26]. Ub commonly modifies protein substrates in the form of a Lys-48– or Lys-11–linked polyUb chain that functions as a signal for proteasome degradation. Besides targeting proteins for degradation, Ub performs many crucial nonproteolytic controlling functions by targeting the substrates with a single Ub moiety or as a polyubiquitin chain with different lysine linkages or a nonlysine-based linear chain [27,28]. Modification of a protein by Ub, called ubiquitination, is catalyzed by a three-enzyme cascade involving Ub-activating enzyme (E1), Ub-conjugating enzyme (E2), and Ub-protein ligase (E3). All E3s have an E2-ubiquitin binding domain that is classified on the basis of the structure of this domain and the mechanism by which they transfer the ubiquitin. For example, the direct transfer of ubiquitin from E2 ubiquitin to its substrate is catalyzed by RING E3s. The catalytic cysteine in HECT (Homologous to E6AP C-Terminus) and RBR E3s (RING-in-Between-RING E3s) takes ubiquitin from E2 ubiquitin and transforms it into an E3 ubiquitin thioester intermediate before transferring it to the substrate [29]. The E3s are the crucial components of the ubiquitination process because they exert strict control on both the efficiency and substrate specificity of the ubiquitination reaction [30]. To make use of the functionality of protein ubiquitination, eukaryotic organisms have produced various Ub ligases. Ub ligases consist of a small number of common catalytic cores, as well as a variety of substrate-recruiting modules and regulatory components. The unique properties of Ub ligases permit them to operate in distinct cellular circumstances, respond to different cellular signals, and process diverse protein substrates [31].

The ubiquitination process plays an important role in the substrate degradation that consequently facilitates the quality and quantity control of proteins, supporting cell homeostasis and ensuring life activities for normal growth. This process is regulated in several ways, including the transcriptional stage, the translational stage by activator or repressor and at the post-translational level [32]. This leads to the specific autophagy of cells by recruiting autophagic adaptors through “eat me” signaling. The initial stage and nucleation steps of autophagy are highly regulated by ubiquitination, meaning that ubiquitination controls the beginning of the autophagy process in response to several stressed conditions [33]. It also controls several other cellular functions, such as intracellular signaling, the growth of the cell, DNA repair, endocytosis, transcriptional regulation, the cell cycle, and apoptosis [34].

Ubiquitin ligases are components of the ubiquitin-proteasome system (UPS) that play a major role in the maintenance of normal cellular metabolism, viability, homeostasis, and cell cycle regulation in response to external stress signals and DNA damage [35]. There are an estimated 600–700 E3 ligase genes representing approximately 5% of the human genome [36]. Ubiquitin ligases can promote the degradation of either oncogenes or tumor-suppressor genes, thus E3s are themselves “druggable” enzymes or serve as potential cancer targets [37,38]. The E3 ubiquitin-ligase enzyme is important for the specific binding of ubiquitin to its target substrate, which depends on their specific domains [39]. In collaboration with ubiquitin-activating enzyme E1 and ubiquitin-conjugating enzyme E2, E3 ubiquitin ligases catalyze the ubiquitination of several biologically important protein substrates for targeted degradation via the 26S proteasome, and they engage in nonproteolytic regulation of their functions and subcellular localization. 

**Figure 2:**
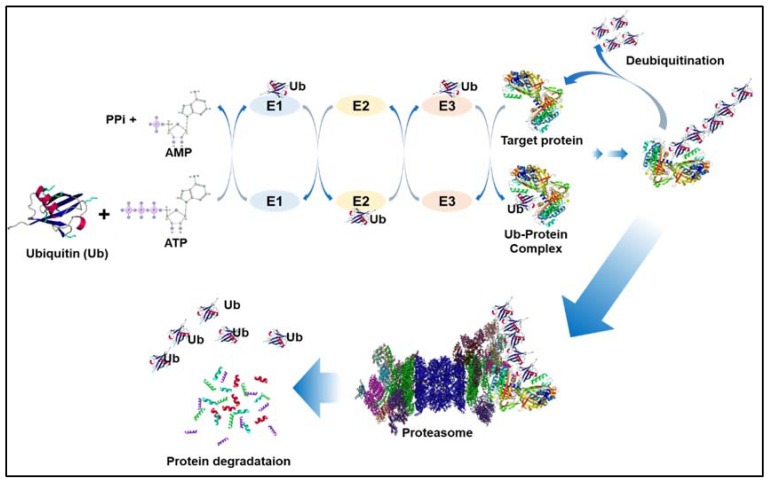
The ubiquitination processes. Ubiquitin (Ub) is added to the selected substrates in three phases, each necessitating the use of three enzymes: E1, E2, and E3. Step 1: The ubiquitin-activating enzyme E1 activates ubiquitin in an ATP-dependent manner. Step 2: Ubiquitin is subsequently transported to one of numerous types of E2, the ubiquitin conjugating enzyme. Step 3: The attachment of ubiquitin to the protein substrate is mediated by one of many E3s. The 26S proteasome recognizes polyubiquinated proteins and degrades them. Small peptides and reusable free ubiquitin are produced by their cleavage. Deubiquitination catalyzes the removal of Ub from substrate proteins.

A general mechanism of the ubiquitination process is shown in Figure 2. The name 26S proteasome was applied in the late 1990s [40]. E3 ubiquitin ligases play a vital role in the regulation of numerous biological processes, such as skeletal muscle atrophy [41]. An increasing body of evidence suggests that aberrant control of some E3 ligases and deubiquitination enzymes is involved in the regulation of lung cancer tumor growth and metastasis [21,42]. They work by ubiquitinating critical signaling nodes in the RAS-RAF-MEK-ERK and PI3K-AKT-mTOR signaling pathways, which govern the biological and metabolic processes of tumor cells [43].

Deubiquitinating enzymes (DUBs), proteases that remove ubiquitin or ubiquitin-like molecules from target proteins or rebuild ubiquitin-chains on target proteins, have recently been recognized as critical regulators of ubiquitination-mediated degradation. DUBs, as a result, have a significant impact on a variety of biological processes and cellular signaling, including the DNA damage response and DNA repair pathways. Therefore, a deeper understanding of how DUBs govern the DNA damage response and DNA repair could lead to new anticancer therapeutic techniques [44].

## 3. Ubiquitination, Deubiquitination and Lung Cancer

In recent years, studies on ubiquitination and deubiquitination have identified that they are involved in the regulation of metabolic reprogramming of cancer cells in various types of cancer [45,46]. The multifaceted mechanism of ubiquitination and deubiquitination is regulated by a specific group of proteases involving approximately 100 deubiquitinating enzymes (DUBs) that are essential for almost all of the biological processes in the cell [38,47,48]. It was discovered that ubiquitination could modify intracellular histones, and thus ubiquitination emerged as a new molecule involved in post-translation modification. Various DUBs are associated with lung cancer. A few of them are listed in Table 2, along with their mode of action.

Ubiquitin-specific protease 22 (USP22), a deubiquitinating enzyme, is a therapeutic target in cancer patients. USP22 activates multiple EGFR downstream signaling pathways, including the STAT3, AKT/mTOR, and MEK/ERK pathways, in lung ADC (adenocarcinoma) cell lines H1975 and PC9, and it stabilizes EGFR protein expression [60]. Sun et al. revealed that the USP36 level was upregulated in a subset of human lung cancers. Their study also demonstrated that USP36 interacts with and deubiquitinates c-Myc in the nucleolus, subsequently stabilizing c-Myc expression [56]. USP37 expression is positively linked with c-Myc protein expression in human lung cancer tissues, indicating that it is considerably elevated. It regulates the stability of c-Myc [57]. USP39 influences lung cancer growth by modulating the intracellular Akt, mTOR, p53, and PARP signaling pathways. As a result, in preclinical and clinical research, USP39 may serve as a potential therapeutic target for the treatment of human lung cancer [58]. It was observed that the expression of USP39 was remarkably elevated in large cell lung carcinoma [58,61]. The emerging roles of USP28 in cancer pathways have been discovered by some recent studies. The oncoprotein c-MYC (deregulation of the c-Myc protein is associated with cancer progression, including in lung cancer) is strongly linked to poor patient survival [62] and is stabilized by USP28 [63,64]. The involvement of USP28 in NSCLC was established by Zhang et al. (2015). They found that increased USP28 mRNA and protein expression was associated with a low patient survival rate [65]. As a result, USP28 expression in NSCLC can be used to predict the outcome of the disease. Upregulation of USP28 also promoted NSCLC cell proliferation and vice versa [65]. A recent study by Ruiz et al. (2020) showed that treatment with a USP28 inhibitor caused a significant drop in c-Myc protein levels, which led to significant regression of murine LSCC tumors and human LSCC xenografts, phenocopying the impact seen with genetic deletion [66]. It was also discovered that USP28 causes NSCLC by activating the STAT3 signaling pathway, which is tightly regulated in mammalian cells, and that overexpression or hyperactivation of this pathway is required for carcinogenesis [67]. 

E3 ubiquitin ligases attach a ubiquitin moiety to target proteins for proteasome-dependent degradation. Mutations of the E3 ubiquitin gene or anomalous expression of E3 ubiquitin ligases could lead to cancer development [68]. The mammalian ubiquitination factor E4B (UBE4B/UFD2a) is an E3 ubiquitin ligase located on chromosome 1p. UBE4B plays an important role in repairing DNA double-strand breaks (DSB), and it also acts as a regulator of p53, a basic effector molecule of the DNA damage response and repair (DDR/R) pathway. Ubiquitin-protein ligase E3C (UBE3C)-mediated ubiquitination is involved in sustaining the CSC (cancer stem cells) properties of NSCLC. In stem-like NSCLC cells, overexpression of UBE3C acted as a stemness enhancer. In vivo and in vitro, knocking out UBE3C reduced NSCLC stemness and carcinogenesis [69]. CBLC, a member of a CBL protein family, has E3 ubiquitin ligase activity that assists activated receptor tyrosine kinases. CBLC has been reported to be an epigenetically demethylated target, and its expression level in NSCLC can be increased after treatment with the DNA methylation inhibitor azacytidine. Immunoblotting and qRT-PCR based studies demonstrated that CBLC is expressed in a variety of lung cancer cells (50%), including lung adenocarcinoma (LUAD) cell lines and a lung squamous cell carcinoma cell line [70]. Pirh2 has a RING-finger domain that has intrinsic activity as an E3 ubiquitin ligase and it directly binds to p53 and induces its degradation [71]. Pirh2 binds to p53 independently of MDM2 and causes the ubiquitination of p53 [72]; therefore, Pirh2 protein adversely regulates p53 [73] and causes lung cancer. It has been reported that Pirh2 enhances the oncogenic properties of human NSCLC carcinoma cells [74]. 

In human malignancies, members of the HECT type E3 subfamily are frequently dysregulated. Cancer formation and chemoresistance are linked to mutations, abnormal expression, and the uncontrolled activity of these enzymes. HECT-type E3s play a role in cancer development by controlling the ubiquitination of the substrates that have either antitumor or protumor roles [75]. HUWE1, UBR5, and TRIP12 are other HECT subfamilies. DUF908 and DUF913, two functional domains at the N-terminus of HUWE1, are comparable to the domain in the Saccharomyces cerevisiae HECT ligase Tom1 and are followed by the ubiquitin-related UBA domain. HACE1 (HECT domain and ankyrin repeat-containing E3 ubiquitin-protein ligase) can be targeted to bind to p53 and a variety of other substrates implicated in carcinogenesis, and it has attracted a lot of interest as a potential anticancer treatment target. UBR5 is expressed in a wide range of cell types. UBR5 is a cellular signal regulator that is involved in a variety of cancer biological processes. The processes of UBR5 in carcinogenesis and development, however, are unknown [76]. 

## 4. Ubiquitination and Deubiquitination-Mediated Therapy for Lung Cancer

Several studies have revealed that cancer cells are very reliant on a functional UPS system for tumor initiation, metabolism and survival [32,46,77,78]. Thus, components of the UPS have attracted attention in the treatment of cancer in the past few decades. UPS as a therapeutic target of cancer has proven successful in multiple myeloma using a proteasome inhibitor called bortezomib [79]. Bortezomib (PS-341) was approved by the FDA for the treatment of multiple myeloma. It has been evaluated in a variety of NSCLC models in vitro and in vivo, and it has been shown to be effective against NSCLC cells [43]. 

Among the three enzymes involved in the ubiquitination system, E3 ligases primarily determine substrate specificity [80,81]. E3 ligases were shown to control the stability and functions of many key regulatory proteins and subsequently regulate a number of cellular processes, including cell proliferation, cell cycle arrest, and apoptosis. E3 ligases are the second most common cancer-related functional gene family after protein kinases, and deregulation of E3s and their associated ubiquitin network is frequently linked to human disorders, including neurological disease and cancer [78,82]. E3s may also be mutated in cancer, as is the case with c-Cbl, a RING E3 ligase that has been shown to be mutated in NCSLC and plays an essential role in lung tumorigenesis and metastasis [83]. Given the fundamental role and specificity of E3 ligases, they are the most common therapeutic target. Compared to the general proteasome inhibitor bortezomib, which interferes with the entire process of protein degradation, targeting a particular E3 ligase is expected to have better target selectivity, leading to better safety as well as less toxicity [80,81]. A therapeutic target should have a major role in the onset of carcinogenesis and be required for the maintenance of cancer cells, as well as suppressing the apoptotic process of cancer cells and stimulating their development as a potent therapeutic target. Activation and overexpression of a target molecule found to be overexpressed in tumor cells should be directly associated with poor patient survival, and significantly, its inhibition will cause cancer cell apoptosis or suppress tumor development. E3 ligases, or particular components of their complexes (MDM2, IAP, APC/CDC20, and others), are known as oncogenes or tumor suppressors in numerous types of cancer; thus, they may serve as potential cancer targets or are themselves “druggable” enzymes. A list of various therapeutic molecules and their targets are given in Table 3.

MDM2, a RING E3 ubiquitin ligase, is known to control the turnover of the tumor suppressor p53. MDM2 is elevated in many cancers and facilitates the proteasomal degradation of p53. Several MDM2 targeted inhibitors that interfere with the interaction of MDM2 with p53 or block MDM2 expression have been developed for the treatment of lung cancer and others, including neuroblastoma, retinoblastoma, leukemia and melanoma [19,84,85,107]. 

Evidence has shown the pivotal role of the ubiquitin/proteasome pathway in the regulation of apoptosis. Many inhibitors of apoptosis (IAP) proteins, that are key regulators of apoptosis and block apoptosis by both the intrinsic and extrinsic pathways, also have RING finger-dependent E3 activities; thus, they catalyze ubiquitylation and the subsequent proteasome-mediated proteolysis [108]. As critical apoptosis regulators, IAPs are potential therapeutic targets.

Initially, preclinical showed that most of the small-molecule inhibitors targeting E3 ligases have antitumor activity, and are likely to be less toxic than chemotherapy. Additionally, clinical trials are being conducted to test their efficacy in combination with conventional anticancer drugs in patients. Although E3 ligases are considered to be promising therapeutic targets, due to limited understanding of ligase–substrate relationships and their biological function, efforts for developing therapeutics targeting E3 ligases have so far been relatively ineffective, with no drugs approved for clinical use. Additional studies are required for better comprehension of the precise mechanism of E3 ligase-mediated substrates and whether some of these ubiquitin molecule targeted therapies can be combined for cancer therapy.

The UPS involves a series of enzymatic processes that use four different enzyme families: E1, E2, E3, and E4. Since the UPS is a critical regulator of the cell cycle, and abnormal cell-cycle regulation can lead to oncogenesis, it is a promising target for new anticancer drugs [109]. Cell signaling responses are crucial in controlling cell characteristics. The primary control nodes serve as a signaling switch that mediates cell processes. Meanwhile, the ubiquitination mechanism controls how these signaling pathways are activated and deactivated. In lung cancer, ubiquitin regulates the PI3K-AKT-mTOR and RAS-RAF-MEK pathways, and ubiquitination of these signaling nodes coordinates cell signal transduction positively or negatively. 

E2 enzymes act as “Ub carriers” and, in some cases, E2s has been shown to be essential for Ub-substrate specificity in many ubiquitination events [110,111]. Numerous E2 enzymes (including CDC34, UBC9, UBE2C, UBE2D, UBE2N, UBE2S, and UBE2Z) have been shown to be significantly overexpressed in lung cancer, contributing to facilitating cell proliferation and tumor growth [38]. Thus, targeting E2s is also a promising strategy for lung cancer treatment. Many small-molecule inhibitors of E2 enzymes have been developed and have shown remarkable efficacy in suppressing cell proliferation in vivo and tumor growth in vivo [38]. CC0651, a small molecule inhibitor that selectively inhibits CDC34, potently suppresses cell proliferation of several human cancer cell lines; however, there is still a lack of preclinical or clinical information on CC0651 in lung cancer [112]. Zhang et al. also revealed that CDC34 positively regulates EGFR-mediated oncogenic signaling in lung cancer, and a PROTAC (proteolysis-targeting chimeras) strategy can be employed to hook small molecules to cullin-RING ligase to directly target CDC34 for degradation [113].

In addition, the E1 enzymes are responsible for initiating the protein degradation process and they play an important role in tumorigenesis. Thus, development of compounds targeting the E1 enzymes has become an increasingly attractive approach. 

TAK-243 (formerly known as MLN7243), which is the first-in-class inhibitor of the UAE, has been shown to cause depletion of cellular ubiquitin conjugates, resulting in a disruption of signaling events, induction of proteotoxic stress, and impairment of cell cycle progression and DNA damage repair pathways. TAK-243 treatment resulted in the depletion of cellular ubiquitin conjugates, subsequently causing the disruption of signaling events in primary human xenografts [20]. Furthermore, combination treatment of TAK-243 and the current SCLC standard therapies may have the potential to improve the efficiency of treatment for SCLC [114].

Another strategy to inhibit the UPS system is targeting DUB activity, which catalyzes the removal of Ub from substrate proteins. Currently, USPs have emerged as a promising therapeutic target class. Among numerous small molecules targeting DUB, the top inhibitors, Pimozide and GW7647, inhibited USP1/UAF1 noncompetitively and displayed selectivity against a number of deubiquitinases. It has been also shown that the USP1/UAF1 inhibitors act synergistically with cisplatin in inhibiting cisplatin-resistant NSCLC cell proliferation [50,108].

Given this, inhibition of the ubiquitin system, including the proteasome, E1, E2, E3 and DUB, has been proven an effective treatment in multiple malignancies including lung cancer. Ubiquitylation is a fundamental process and unusual functions or the abnormal regulation of ubiquitylation enzymes are involved in various disorders, including cancer [23]. The regulation of ubiquitylation enzyme activity is likely to provide strategies for therapeutic interventions [93] and targeting the ubiquitin–proteasome system is an effective therapy for cancer treatments [115]. 

Inhibition of E3 ligase established a potent therapeutic target in the treatment of lung cancer [96]. Additional analysis and a better understanding of the UPS would undoubtedly produce a slew of new therapeutic targets. Bortezomib provided the precedent for UPS inhibition and its use in cancer treatment. Second-generation proteasome inhibitors, as well as other UPS targets, exist and are in clinical development. The function of selective therapies will only become more valuable as the cellular mechanisms responsible for malignant transformation and propagation are revealed; the UPS clearly contains a number of such selective targets [109]. 

In the future, prospective, small-molecule inhibitors, such as molecular glues and heterobifunctional degraders like PROTACs, could be used to target numerous proteins that were previously thought to be untargetable. PROTACs form a ternary complex with a hijacked E3 ubiquitin ligase and a target protein, causing the target protein to be polyubiquitinated and degraded [116]. This approach has broad applications since it can be used to degrade a large number of proteins that have critical roles in driving cancer.

## 5. Conclusions

Current research in cancer biology has shown that the ubiquitination pathway plays a key role in the regulation of cellular processes, and its dysregulation leads to cancer development. Consequently, the UPS is a valuable repository of specific drug targets for the treatment of cancer. The complexity of the UPS system generates noteworthy challenges, and thus there is a need for further research to investigate the entirety of the ubiquitination and deubiquitination pathways. Additionally, there is also a need for a better understanding of ubiquitination in cancer development, its progression and metastasis, to expand the accessible drug-target space. Focusing on this pathway is a unique, promising approach that will move the field from hypothesis-driven research to clinical applications, lowering cancer-related mortality.

## Figures and Tables

**Table 1:** Comparison between small cell lung carcinoma and nonsmall cell lung carcinoma.

Characteristics	Small Cell Carcinoma	Non-Small Cell Carcinoma
Cell size	Small cell	Larger cell
Location	Peribronchial location	Any part of the lung
Metastasizes/Spread	Fast spread	Slow spread
Fatality	More, if untreated	Less
Type	Two types: small cell carcinoma and combined small cell carcinoma	Adenocarcinoma and squamous cell carcinoma
Diagnosis	In early stage of carcinoma	In late stage of carcinoma
Treatment	Fast	Slow, due to late examination
Incidence of total lung cancer	15%	85%

**Table 2:** Representative DUBs and their associations with lung cancer.

UPS	Target/Function	References
USP1	Involvement in translesion synthesis and DNA damage	[49]
USP7	Ki-67 antigen (Ki-67)	[50]
USP8	Regulate RTKs including EGFR and ERBB2	[51]
USP14	Promotes NSCLC cell proliferation by β-catenin accumulation	[52]
USP17	Deubiquitinates p53 and Mdm2 to alter the stability and activity of p53	[53]
USP22	Promotes tumor progression and induces epithelial-mesenchymal transition (EMT)	[54,55]
USP28	Stabilization of the oncoprotein c-Myc	[11]
USP36	Regulate and stabilize c-Myc	[56]
USP37	Directly stabilizes c-Myc	[57]
USP39	Akt, mTOR, p53, and PARP signaling pathways	[58]
USP44	Akt signalling	[59]

**Table 3:** List of various compounds that target the ubiquitin system by different modes of action in lung cancer.

Compounds	Target	Modes of Action	References
Nutlin-3a	MDM2	Competitively binds to the Mdm2-P53 interaction region, activating the P53 pathway and causing cell cycle arrest, cell death, and growth inhibition.	[19,84,85]
RG7388	MDM2	The derivatives of nutlin-3a inhibit the Mdm2-P53 binding site	[86]
RG7112	MDM2/MDMX	Restoration of p53 activity by inhibiting the p53-MDM2 interaction	[86,87]
ATSP-7041	Dual inhibition of MDM2 and MDMX	Inhibitor of MDM2 and MDMX for p53-dependent processes	[88]
AT-406 (also known as Debio 1143 or SM-406)	XIAP, IAP1 and IAP2	Suppresses the inhibitor of apoptosis protein	[89]
TL-32711	IAP	Suppresses the inhibitor of apoptosis protein	[90]
HGS-1029	IAP2	Suppresses the inhibitor of apoptosis protein	[91,92]
TAK-243 (MLN7243)	UBA1	Causes depletion of cellular ubiquitin conjugates resulting in disruption of signaling events	[93]
NAHA (Novel Hydroxamic Acid-Derivative)	Cdc20	Decreases the expression of Cdc20. Inhibits tumor proliferation in vitro and in vivo associated with the initiation of apoptosis	[94]
Thalidomide	Multiple target Cereblon (CRBN)	Significantly increases PPAR (Peroxisome proliferator-activated receptor) gamma protein expression, significantly increased PPRE (PPAR response element) reporter activity and decreases NFkB reporter activity in LCC (large cell carcinoma) cells	[95,96,97,98]
Pomalidomide	CRBN	Suppresses CRBN E3 activity, reducing c-Myc and IRF4, suppressing MM cell transcriptional activity	[96,99,100]
Lenalidomide	CRBN	Induces apoptosis and modifies gene expression in NSCLC cells	[95,100,101,102,103]
Pimozide and GW7647	USP1/UAF1 complex	Involved in translation synthesis and DNA damage response in NSCLC	[49,104]
b-AP15 (known as VLX1500)	UCHL5 (ubiquitin C-terminal hydrolase 5) and USP14	Induces tumor cell apoptosis and inhibits tumor progression	[43,105,106]
